# Complete Genome Sequence of *Acinetobacter baumannii* CIP 70.10, a Susceptible Reference Strain for Comparative Genome Analyses

**DOI:** 10.1128/genomeA.00850-15

**Published:** 2015-07-30

**Authors:** Thomas Krahn, Daniel Wibberg, Irena Maus, Anika Winkler, Alfred Pühler, Laurent Poirel, Andreas Schlüter

**Affiliations:** a Center for Biotechnology, Bielefeld University, Bielefeld, Germany; b Faculté des Sciences, Université de Fribourg, Fribourg, Switzerland

## Abstract

The complete genome sequence for the reference strain *Acinetobacter baumannii* CIP 70.10 (ATCC 15151) was established. The strain was isolated in France in 1970, is susceptible to most antimicrobial compounds, and is therefore of importance for comparative genome analyses with clinical multidrug-resistant (MDR) *A. baumannii* strains to study resistance development and acquisition in this emerging human pathogen.

## GENOME ANNOUNCEMENT

The emerging human pathogen *Acinetobacter baumannii* has acquired resistance genes with unprecedented rapidity (1). While it was susceptible to most antibiotics about 40 years ago (1), it has had an increasing tendency to develop multidrug resistance (MDR) over the last two decades (2). *A. baumannii* is a remarkably persistent organism causing nosocomial and community-acquired infections, such as septicemia, pneumonia, endocarditis, and meningitis (3).

Here, we present the genome sequence of *A. baumannii* strain CIP 70.10 (ATCC 15151) (4), a human inhabitant originally isolated in France in 1970 (5), which represents an important reference strain, since it is susceptible to most antibiotics. Accordingly, the strain is often used as recipient to study transmissible antibiotic resistance among *Acinetobacter* species.

For sequencing of the *A. baumannii* CIP 70.10 genome, an 8-kb mate pair sequencing library (Nextera mate pair sample preparation kit; Illumina, Inc.) was constructed and sequenced on an Illumina MiSeq system. The sequencing approach produced 2,812,699 sequence reads accounting for 598,180,197 bases of total sequence information. Assembly of the obtained sequence reads using the GS *de novo* Assembler software (version 2.8, Roche) (6, 7) resulted in one scaffold composed of 17 contigs for the 3.9-Mb chromosome of the strain and in another scaffold representing a 7.7-kb cryptic plasmid. Subsequently, a PCR-based gap closure approach (8, 9) was applied to finalize the genome sequence of the strain leading to the circular chromosome (3,928,513 bp) and plasmid (7,742 bp), which featured G+C contents of 38.92% and 37.56%, respectively. Annotation of the genome was performed using the GenDB 2.0 system (10) and resulted in the prediction of 3,607 coding sequences, 71 tRNA genes, six *rrn* operons for the chromosome, and 13 coding sequences for the plasmid.

Genome analyses revealed that strain CIP 70.10 contains different putative virulence and resistance determinants on its chromosome. Among these are genes for siderophore biosynthesis, ferrous iron transport (Feo system), and heme uptake facilitating iron acquisition (11). Moreover, three type I pili systems were identified, which putatively play a role in adherence and biofilm formation (12). Further genes contributing to biofilm formation, such as the *pgaABCD* locus (biosynthesis of poly-β-1-6-*N*-acetylglucosamine) and the AHL (*N*-acyl homoserine lactone synthesis) cluster were identified (12). Additionally, the chromosome harbors genes encoding a type IV pili system, which is supposed to mediate twitching motility and DNA uptake (13), the outer membrane protein *ompA* gene, whose product facilitates adherence to cell surfaces and cell invasion (12), and a type VI secretion system, also involved in promoting cell invasion (12).

Putative antibiotic resistance determinants were identified using ARG-ANNOT (14), CARD (15), and the Resfams database (16). Among others, CIP 70.10 harbors genes encoding the three resistance-nodulation-division (RND) efflux pumps, AdeABC, AdeIJK (17), and AdeFGH (18), and the β-lactamase gene *bla*_OXA-64_. Nevertheless, susceptibility tests indicated that this strain is susceptible to most antibiotics (5). Accordingly, the genome of strain CIP 70.10 can be used as a reference for comparative analyses, e.g., with genomes of MDR *A. baumannii* strains to elucidate the acquisition mechanisms of resistance determinants.

### Nucleotide sequence accession numbers.

This *A. baumannii* CIP 70.10 genome sequence has been deposited at EMBL (EBI)/GenBank under the accession numbers LN865143 (chromosome) and LN865144 (plasmid).
